# Correlation of Vitamin D Deficiency With Severity of Chronic Heart Failure as Assessed by Functional Class and N-Terminal Pro-Brain Natriuretic Peptide Levels

**DOI:** 10.7759/cureus.13522

**Published:** 2021-02-23

**Authors:** Parminder S Otaal, Sudheer Pachipala, Lipi Uppal, Dinakar Bootla

**Affiliations:** 1 Department of Cardiology, Post Graduate Institute of Medical Education and Research, Chandigarh, IND; 2 Department of Medicine, Post Graduate Institute of Medical Education and Research, Chandigarh, IND

**Keywords:** heart failure, severity, nt-pro bnp, vitamin-d deficiency

## Abstract

Introduction

Chronic heart failure (CHF) is a major cause of mortality and morbidity in spite of tremendous advances in medical therapies. Vitamin D deficiency has been increasingly recognised in heart failure and its therapeutic as well as prognostic implications are debated. This study was carried out to examine the relationship of Vitamin D levels with severity of heart failure as assessed by NYHA functional class and serum N-terminal pro-brain natriuretic peptide (NT-pro-BNP) levels in vitamin D deficient patients with CHF.

Methodology and results

In this cross-sectional analysis, 119 patients of symptomatic CHF presenting to the outpatient/inpatient department of cardiology in a tertiary care institute in North India were screened. Patients were categorised according to their functional class as New York Heart Association (NYHA) class II, III, IV and their serum levels of vitamin D and NT-pro-BNP were measured. Out of 119 patients, 107 (90%) were found to have low vitamin D levels which were classified as insufficient (20-30 ng/ml) (n=25, 23%) or deficient (<20 ng/ml) (n=82,77%). The mean NT-pro-BNP levels increased significantly across functional class as 3783±6132 pg/ml, 7866±4383 pg/ml, 21115±11905 pg/ml in NYHA class II, III and IV respectively (p=0.000). The respective mean serum Vitamin D3 levels of 11.6±5.8ng/ml, 12.2±7.9 ng/ml, 14.4±8.9 ng/ml were not significantly different between classes (p=0.234). We found no correlation between serum NT-pro-BNP and serum vitamin D levels in the study cohort across various NYHA classes. In multivariate regression model, after adjusting for various co-variates, vitamin D levels were not significantly associated with NT-pro-BNP or functional class in patients with CHF.

Conclusion

Patients with CHF have a high prevalence (90%) of vitamin D deficiency. Although NT-pro-BNP levels increase significantly, vitamin D levels do not vary significantly with worsening NYHA classes. Further, no consistent significant correlation of vitamin D deficiency with NT-pro-BNP across different NYHA classes was observed. Thus, low levels of vitamin D didn’t predict the severity and prognosis of patients with heart failure.

.

## Introduction

With growing older population and improved overall survival, the prevalence of chronic heart failure (CHF) is increasing and currently estimated at about 37.7 million across the world [1]. Even with optimal medical management and recent therapeutic advances, the prognosis of heart failure remains dismal with 60% to 70% mortality within five years of follow-up [2]. The pathophysiological mechanisms implicated for poor outcomes include excessive neurohormonal activation, negative cardiac remodelling, persistent inflammation, fluid retention and deficiency of various macronutrients and micronutrients [3]. Vitamin D or 1,25 di-hydroxy cholecalciferol deficiency particularly seems to influence cardiovascular outcomes including myocardial infarction, stroke, peripheral vascular disease and heart failure [4,5] . The exact mechanism remains obscure, however in vitro studies have demonstrated its role in intracellular calcium metabolism, myocardial contractility, downregulation of renin-angiotensin-aldosterone system, prevention of excessive cardiac remodelling and suppression of inflammation [6,7]. Genetic disruption of the vitamin D receptor in animal studies has led to overstimulation of the renin-angiotensin system, in turn causing high blood pressure and increased concentration of biomarkers like atrial natriuretic peptide [8]. Some of the previous studies have shown worse prognosis with vitamin D deficiency in those with heart failure [9,10], although data of its effect on the severity of heart failure are lacking. So, we hypothesized that low vitamin D levels may contribute to the pathogenesis and severity of heart failure and can become a potential therapeutic target in such patients. Amino terminal pro-brain natriuretic peptide (NT-pro-BNP) which is an inactive derivative of prohormone pro-BNP, is a reliable marker of mortality and prognosis in heart failure [11]. It objectively assesses the severity of heart failure and is often used in therapeutic monitoring in such patients [12]. Because of its proven reliability, we used NT-Pro-BNP in addition to the functional NYHA class to objectively assess heart failure severity. We, therefore, conducted this study to examine the degree of hypovitaminosis-D in CHF and to assess any correlation with the severity of heart failure. 

## Materials and methods

Patient selection

This is a single centre, cross-sectional study conducted by enrolling heart failure patients presenting to the outpatient section or admitted in the inpatient section of Advanced Cardiac Centre of Department of Cardiology in the Postgraduate Institute of Medical Education and Research, a tertiary care hospital in North India, from July 2016 to November 2017.

Inclusion criteria

Patients more than 18 years of age with symptoms of heart failure (New York Heart Association, NYHA class II, III and IV) for more than three months duration, left ventricular ejection fraction <45%, and serum 25(OH)D3 < 30 ng/ml were included.

Exclusion criteria

Patients on vitamin and calcium supplements, those suffering from systemic diseases including chronic kidney disease, malignancy, sarcoidosis, gastro-intestinal malabsorption states and untreated valvular heart disease were excluded from the study.

The study conforms to the Helsinki declaration. Ethical approval was taken from the Institute’s ethic thesis committee. Written informed consent was given by all patients. 

Biochemical investigations

All the patients underwent baseline blood investigations including serum electrolytes, serum creatinine, complete blood counts including haemoglobin levels. Serum 25-hydroxy vitamin D3 (25(OH)D3) and N-terminal pro-brain natriuretic peptide (NT-pro-BNP) levels were measured in all the patients after overnight fasting. Vitamin D deficiency was defined as 25(OH)D3 levels < 20 ng/mL, insufficiency as 20-29 ng/mL and sufficiency as ≥30 ng/mL [13].

Cardiac evaluation

The participants were classified according to New York Heart Association (NYHA) functional classification into class II, III and IV [14]. 2D echocardiography was performed in all participants by an experienced cardiologist who was blinded to patient characteristics. Left ventricular ejection fraction (LVEF) was calculated by Simpson’s biplane method and left ventricular dimensions were measured in M-mode using Philips IE33/ Philips EPIQ 7 (echocardiography) system (Philips healthcare TM, Amsterdam, the Netherlands) [15]. 

Statistical analysis

Normally distributed parameters were presented as mean ± standard deviation. Continuous parameters following a non-normal distribution were presented as median and interquartile range (IQR). Categorical data were given as percentages. Based on the patients' worst NYHA functional class in the last three months, three subgroups were formed, NYHA II, NYHA III and NYHA IV. Comparisons among heart failure subgroups were performed by analysis of variance (ANOVA) with P for linear trend for continuous parameters or ANOVA on ranks for nonparametric data. A chi-square test was performed for categorical variables. Simple correlation analyses (Pearson or Spearman correlations where appropriated) and multiple linear regression analyses including several independent variables were performed to examine whether vitamin D levels were associated with NT-pro-BNP levels. A p-value < 0.05 was considered statistically significant. Data analysis was done using Statistical Product and Service Solutions (SPSS) 22.0 statistical package.

## Results

Baseline characteristics

Baseline characteristics of the study population are shown in Table 1.

**Table 1:** Baseline characteristics of the study population according to NYHA class. T2DM: type 2 diabetes mellitus; HTN: hypertension; CAD: coronary artery disease; BMI: body mass index; TG: triglyceride; HDL: high-density lipoprotein; LDL: low-density lipoprotein; TSH: thyroid-stimulating hormone; LVEF: left ventricular ejection fraction; Hb1Ac: glycosylated hemoglobin; NYHA: New York Heart Association.

Parameter	NYHA II (n = 39)	NYHA III (n = 29)	NYHA IV (n = 39)	TOTAL (n = 107)	p-value
Age (years)	59.21±12.3	56.62±14.66	56.97±11.24	57.69±12.55	0.64
Female, n (%)	12 (30.7)	8 (27.5)	12 (30.7)	32 (29.9)	0.95
Male, n (%)	27 (69.2)	21 (72.4)	27 (69.2)	75 (70.09)	
Comorbidities					
T2DM, n (%)	11 (28.2)	14 (48.2)	23 (60)	48 (44.8)	0.02
HTN, n (%)	14 (35.8)	16(55.1)	22 (56.4)	52 (48.5)	0.137
CAD, n (%)	29 (74.3)	21(72.4)	30 (77)	80 (74.7)	0.912
Smoker, n (%)	12 (30.7)	11 (38)	13 (33.3)	36 (33.6)	0.575
Alcohol, n (%)	15 (38.4)	14 (48.2)	14 (35.8)	43 (40.1)	0.567
BMI (kg/m^2^)	26.6±3.49	25.64±3.58	26.82±3.81	26.42±3.63	0.385
TG (mg/dL)	110.61±46.96	108.04±31.23	112.46±45.64	110.59±42.4	0.91
HDL (mg/dL)	38.56±8.33	31.11±9.64	38.94±10.96	36.68±10.21	0.002
LDL (mg/dL)	83.44±25.76	73.69±27.66	85.87±30.54	81.68±28.27	0.191
TSH (mU/L)	3.75± 1.78	3.156±1.48	3.93±2.37	3.46±1.95	0.442
Number of patients with previous decompensations, n (%)	4 (10.2%)	5 (17.2%)	6 (15.3%)	15 (14.01%)	0.681
LVEF (%)	34.49 ± 6.76	27.45 ± 10.21	27.13 ± 7.49	29.9± 8.73	0.0001
HbA1C (%)	6.12 ± 1.21	6.7 ± 2.01	7.31 ± 1.83	6.71 ± 1.74	0.01

Out of a total of 119 patients with CHF screened, 12 had normal 25(OH) vitamin D3 levels and were excluded from the study. Rest 107 (90%) patients were categorised according to NYHA classification as NYHA II (n=39), III (n=29) and IV (n=39). The mean age was 57.69±12.55 years with males comprising 70% of the total study population. The prevalence of risk factors included type 2 diabetes mellitus in 48 patients (44.8%), hypertension in 52 patients (48.5%), history of smoking in 36 patients (33.6%), history of alcohol abuse in 43 patients (40.1%). Eighty out of 107 (74.7%) patients had a history of coronary artery disease. There was no difference in the prevalence of individual risk factors among the three groups except type 2 diabetes which was more commonly seen in class IV (p=0.02). Mean LVEF was 34.49±6.76%, 27.45 1±0.21% and 27.13± 7.49%, respectively, in class II, class III and class IV, respectively (p=0.001).

**Figure 1:**
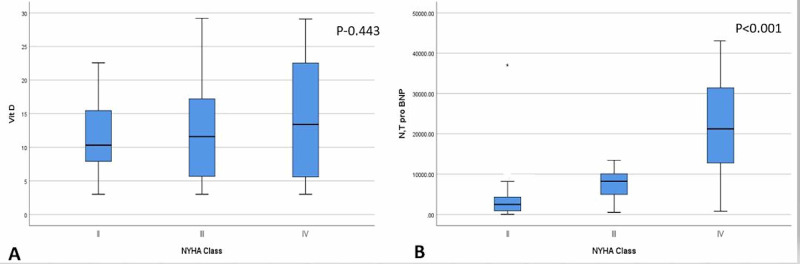
Vitamin D levels (panel A) and NT-pro-BNP levels (panel B) in NYHA class II, III and IV of chronic heart failure. NYHA: New York Heart Association Class; Vit D: Vitamin D level; NT-pro-BNP: N-terminal pro-brain natriuretic peptide.

Serum 25(OH)D3, NT-pro-BNP levels and NYHA class: Of 107 patients included in the study, 82 patients (76.63%) had 25(OH) D3 deficiency (<20 ng/ml) and 25 patients (23.36%) had 25(OH) D3 insufficiency (20-30 ng/ml) with mean levels of 11.6±5.8, 12.2±7.9, 14.4±8.9 ng/ml in class II, III and IV, respectively (Figure 1, panel A). Overall, these levels were not significantly different among the three groups (p=0.234). However, NT-pro-BNP levels increased significantly with 3783.34±6132.36 pg/ml, 7866±4383.3 pg/ml and 20670.31±12072.28 pg/ml in class II, III and IV with p<0.001 (Figure 1, panel B). So, although increasing NT-pro-BNP levels have a significant correlation with worsening NYHA class, vitamin D levels don’t. 

**Figure 2:**
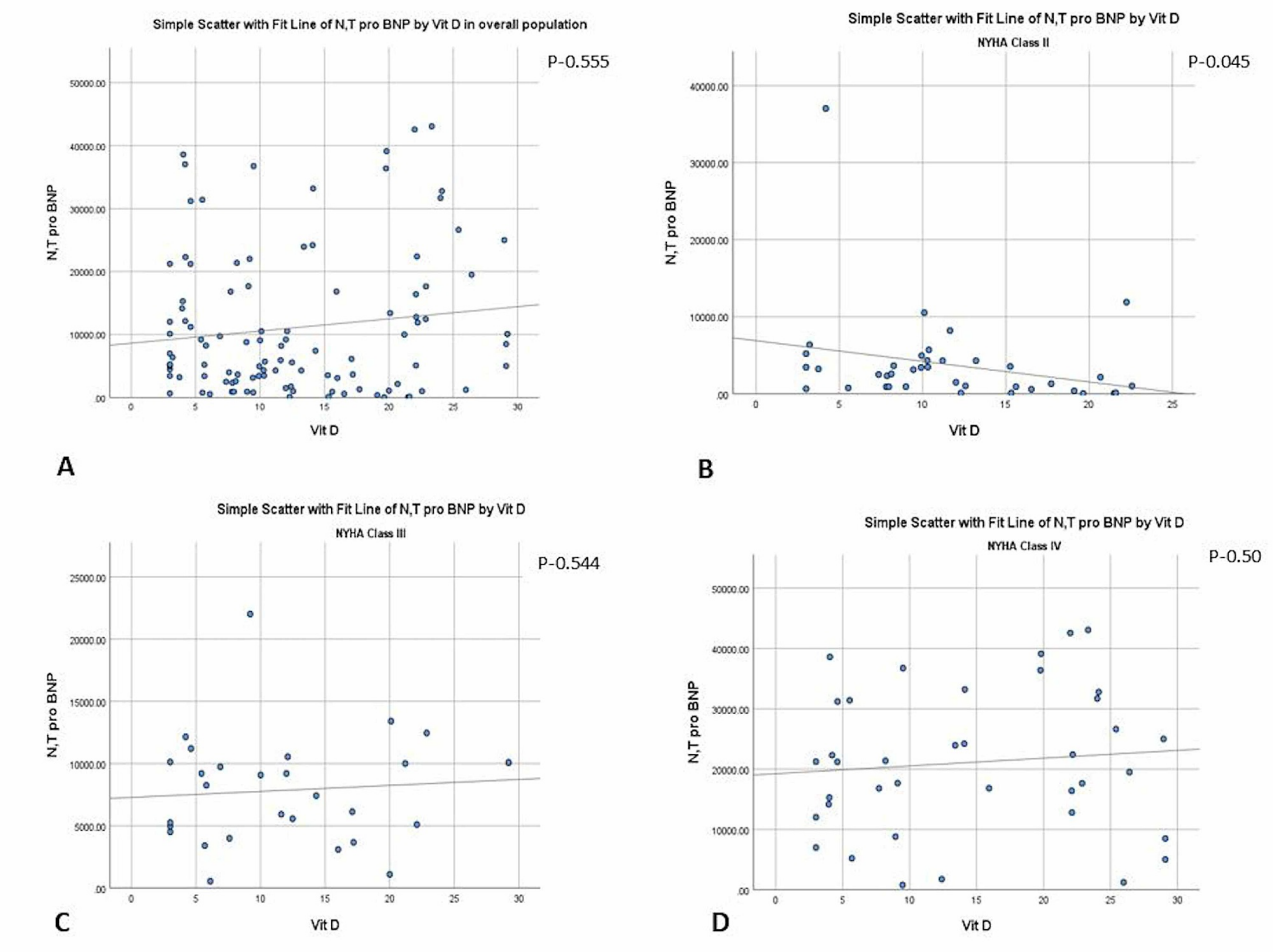
The correlation of NT-pro-BNP levels with vitamin D levels in the overall study cohort (panel A) and in patients in NYHA class II (panel B), III (panel C) and IV (panel D). NT-pro-BNP: N-terminal pro-brain natriuretic peptide; NYHA: New York Heart Association.

Correlation of vitamin D levels with NT-pro-BNP: No significant correlation was observed between serum NT pro BNP and serum vitamin D levels in the overall study cohort or among most classes of heart failure. However, among patients with NYHA class II, an inverse significant correlation was seen with r=-0.323, p=0.045, meaning thereby that low levels of vitamin D were associated with significantly higher NT-pro-BNP levels (Figure 2). NT-pro-BNP levels did not vary among patients with severe vitamin D deficiency (<15ng/ml) when compared to those with higher levels (15-30 ng/ml) [9172.5±10080 pg/ml vs 12685±13133pg/ml; p=0.174]. In the multivariable regression model, after adjustment for various univariates, no significant correlation was observed (B=-41.386 95% CI -262-179; p=0.711).

 

 

## Discussion

Numerous longitudinal and cross-sectional studies have emphasized the importance of low vitamin D levels in population to predict the risk of cardiovascular disease and outcomes [4,5,9,10,16]. Initial population-based studies like National Health and Nutritional Examination Survey (2001-2004) provided the evidence of hypovitaminosis (serum vitamin D3 levels <30ng/ml ) in 74% of the enrolled candidates with cardiovascular diseases including coronary artery disease, heart failure, and peripheral artery disease [17]. Similar findings were mirrored in the Framingham offspring study where individuals with 25(OH)D3 levels < 37.5 mmol/L had a hazard ratio of 1.62 for the development of cardiovascular diseases compared to individuals with levels >37.5 mmol/L [5]. Interestingly, few other studies have noted an increased incidence of cardiovascular events during winter months, a finding which correlates with low vitamin D levels owing to reduced sun exposure [18].

In the setting of heart failure, some authors [19-21] have described the increased prevalence of vitamin D deficiency. However, its relationship with severity and causality remains unknown. Alsafwah et al. reported a very high prevalence of vitamin D deficiency in those with heart failure (95%) than in the normal population (33%); however, no difference was observed between compensated and decompensated heart failure patients [19]. In a large study comprising of 23,793 vitamin D deficient patients from the Intermountain Health care system, the hazard ratio for development of heart failure in patients with very low vitamin D (< 15 ng/ml ) was 2.01 vs 1.31 in patient with low vitamin D levels (16-30ng/ml) [16]. The increased incidence and prevalence of heart failure in patients with vitamin D deficiency points towards its protective effects via various local or systemic mechanisms and thus has been explored further [22].

Our study found a very high prevalence (90% of those screened) of vitamin D deficiency or insufficiency in those with CHF. Indians are known to have vitamin D deficiency in general population ranging from 50% to 94% and the causes include poor dietary intake, inadequate sunlight exposure and increased skin pigmentation [23]. Additionally, in patients with heart failure inadequate gastrointestinal absorption, decreased mobility and persistent catabolic state may further contribute. Nonetheless, even after adjustments for physical limitation, studies evaluating the cardiovascular outcomes in CHF have reported poor prognosis in patients having vitamin D deficiency [9].

The present study further evaluated the prognostic role of vitamin D levels and its correlation with serum NT-pro-BNP levels in patients with CHF. Although vitamin D levels were insufficient or deficient in 90% of patients, it had no significant correlation with clinical severity of heart failure or NT-pro-BNP levels. Although NT-pro-BNP in our study progressively increased with mean serum levels of 3783.34±6132.36, 7866±4383.3 and 20670.31±12072.28 pg/ml in patients in NYHA class II, III and IV, respectively (p< 0.001), no significant trend was observed in vitamin D levels. Additionally, NT-pro-BNP levels did not differ among patients with severe vitamin D deficiency (<15ng/dl) vs those with low to moderately low levels (15-30ng/ml) [9172.5±10080 pg/ml vs 12685±13133pg/ml; p=0.174]. 

Other studies examining the effects of severe vitamin D deficiency and its supplementation have reported contradictory findings. Our results are supported by a randomised controlled trial by Seirafian et al. in which in spite of adequately replacing vitamin D in dialysis-dependent renal failure patients, there was no significant difference in serum pro-BNP, iPTH, calcium, phosphorus or albumin levels after 12 weeks between study groups [24]. Another study by Schleithoff et al. reported improvement in cytokine profile including TNF alpha and Interleukin 10; however, no change was noted in the NT-pro-BNP levels or clinical outcome in those who received vitamin D supplementation [25]. Some other authors also did not find any correlation between NT-pro-BNP and vitamin D levels in patients with congenital heart disease or myocardial infarction [25,26].

A few observational studies, like the one by Liu et al. described an association between low vitamin D levels and activated renin-angiotensin system and proinflammatory state. The study found increased plasma renin activity and higher levels of C reactive protein and TNF alpha in those with low vitamin D levels. They also noted a numerically increasing rate of hospitalisations, across decreasing 25(OH) tertiles [10]. Similarly, in an observational study by Schierbeck et al. in a population of 148 heart failure patients with reduced ejection fraction, low levels of vitamin D and secondary hyperparathyroidism were associated with increased mortality during 3.5 years of follow-up. Moreover, NT-pro-BNP levels increased significantly with increasing PTH tertiles [27]. Thus most randomised controlled trials have been unable to conclusively define the role of vitamin D in patients with heart failure [28]. VINDICATE study suggested that vitamin D might offer cardio protection as it found an increase of 6% in LVEF after supplementing with 4,000 IU of vitamin D daily for six months. Despite this, it did not find any difference in 6 min walk test (the primary endpoint) or in NT-pro-BNP levels [29].

Our study presents another piece of evidence supporting no correlation of serum vitamin D levels with severity of heart failure as measured by NYHA classification and serum NT-pro-BNP levels, in the Indian population. A recent metanalysis also reached a similar conclusion that vitamin D supplementation has a small effect on circulating inflammatory markers but no improvement in serum NT-pro-BNP levels or left ventricular ejection fraction [30]. 

Just like its ambiguity as a marker of poor prognosis, vitamin D continues to suffer from the lack of evidence demonstrating its protective effects and therapeutic potential. In the present analysis in patients of chronic heart failure with reduced ejection fraction, severity of vitamin D deficiency did not influence functional severity and is unlikely to affect prognosis.

However, the present study has several limitations. We didn’t measure serum calcium and serum parathormone levels. We understand that vitamin D deficiency leads to low calcium and secondary hyperparathyroidism which is known to influence heart failure outcomes. Moreover, only heart failure patients with reduced ejection fraction were included in our study and results cannot be extrapolated to those patients presenting with heart failure with preserved ejection fraction. Additionally, as the sample size is small, a larger study is needed to validate our findings.

## Conclusions

Patients with CHF were found to have a high prevalence of vitamin D deficiency. Worsening of levels of vitamin D deficiency were not associated with increasing severity of CHF as measured by NT-pro-BNP or functional NYHA class in patients with reduced ejection fraction. Further low levels of vitamin D were not associated with prognosis of heart failure.
